# Patient Concerns Inventory for Arabic Patients with Head and Neck Cancer: A Cross-Cultural Adaptation and Preliminary Validation

**DOI:** 10.3390/curroncol33010012

**Published:** 2025-12-24

**Authors:** Abdullah M. Alsoghier, Bader A. Alwhaibi, Abdullah F. Alnuwaybit, Simon N. Rogers, Saif A. Aljabab

**Affiliations:** 1 Department of Oral Medicine and Diagnostic Sciences, College of Dentistry, King Saud University, Riyadh 12372, Saudi Arabia; 2 College of Medicine, King Saud University, Riyadh 12372, Saudi Arabia; 438105841@student.ksu.edu.sa (B.A.A.); 438104215@student.ksu.edu.sa (A.F.A.); 3 Oral and Maxillofacial Department, Wirral University Teaching Hospital, Arrowe Park, Liverpool CH49 5PE, UK; simon.rogers10@nhs.net; 4 Radiation Oncology Unit, College of Medicine, King Saud University, Riyadh 12372, Saudi Arabia; saljabab@ksu.edu.sa

**Keywords:** head and neck neoplasms, communication, patient reported outcome measures, health-related quality of life, validity and reliability

## Abstract

Introduction: Head and neck cancer (HNC) treatments often lead to significant post-treatment side effects that affect patients’ quality of life. This study aimed to translate and validate the post-treatment Patient Concerns Inventory for head and neck (PCI-HN) into Arabic among HNC survivors. Methods: This study employed a cross-sectional design, where PCI-HN was translated and assessed for content and face validity by clinical experts and patients, respectively. Revisions to multiple items related to ‘social and religious welfare’. Patients’ responses were then analysed to assess internal consistency (Cronbach’s alpha) and test–retest reliability (Cohen’s Kappa). Results: Thirty-eight participants (19 males, 19 females, mean age 50.68 ± 16.13 years) were included. The Arabic PCI-HN demonstrated good overall internal consistency (α = 0.723) but fair test–retest agreement (κ = 0.22), likely reflecting dynamic changes in HNC post-treatment experiences. Conclusion: The Ar-PCI-HN can be a helpful instrument for capturing distinct aspects of the survivorship experience among Arabic-speaking HNC survivors. Determining the clinical interpretability and ability to detect changes over time requires further multi-centre and multi-country clinical studies. This would be necessary to ensure its integration into routine outpatient consultations for Arabic-speaking patients in Arab countries and globally.

## Simple Summary

Individuals with head and neck cancer usually experience many concerns related to their diagnosis and care needs. Examples could include the treatment of undesired effects on physical, psychological and social aspects. There is no available instrument to investigate the problems associated with this condition for Arabic patients. The present study translated a commonly used patient concern inventory tailored to this condition and its treatment. It tested its precision and accuracy to measure individual preferences and needs. There were varying cultural views on topics such as social and religious issues between the original English and its Arabic translation. Recommendations were presented for further testing with large patient populations and multiple hospitals across the Arabic countries.

## 1. Introduction

Head and neck cancers (HNC) constitute a heterogeneous group of malignancies that significantly affect basic human functions, including speech, taste, swallowing, breathing, and appearance. Globally, HNC accounts for more than 890,000 new cases and over 450,000 deaths annually, making it the seventh most common cancer type [1]. The oral and oropharyngeal cancer figures, based on 448 million individuals living in 23 countries of the Middle East and North Africa region, showed an age-standardised incidence rate of 2.6 and 1.1 per 100,000 population for males and females, respectively [2]. Incidence rates for HNCs are increasing and expected to double or increase by 2–4 times by 2040 in the Gulf Cooperation Council countries, including Saudi Arabia, Bahrain, Oman, Kuwait, Qatar, and the United Arab Emirates [3]. It is also notable that variations exist between regions within the same country, where sometimes younger adults and females are unexpectedly at higher risk [3,4].

Survivors of HNC frequently report late and long-term effects and concerns, such as xerostomia, dysphagia, pain, fatigue, anxiety, and body image [5]. These can have a profound impact on physical health, emotional stability, social interactions and overall health-related quality of life [6]. Such concerns are often underreported or missed in routine consultations, as clinical care often focuses on tumour control and survival metrics.

Given the physical and functional impairment combined with the psychosocial impact of HNC and its treatment, a shift toward holistic and patient-centred clinical care has become essential. To help achieve this, patient-clinician consultations benefit from tailored information based on patients’ concerns. This can enhance patient satisfaction and potentially improve care outcomes [7]. Previous work on patients with oral precancerous lesions demonstrated that a somewhat discordance concerning patient-clinician agreement on important topics is essential to identify what exactly a patient would like to know about their disease, address their concerns (e.g., fear of cancer recurrence or progression) and determine which aspects of their disease they prioritise over others [5].

The Patient Concerns Inventory for Head and Neck (PCI-HN) is a patient-reported outcome measure tool that facilitates patient-focused consultations for various diseases, including HNC [5]. This tool simply asks the patient to choose one or more of the 56 topics they wish to discuss during clinical consultations across four domains: physical and functional well-being; social care and social well-being; psychological, emotional, and spiritual well-being; and treatment-related issues [5]. This enables patients to identify issues they wish to discuss with the clinician during routine clinical consultations.

Furthermore, PCI-HN demonstrated excellent content validity compared to other instruments addressing HNC-related unmet needs, including physical and psychological needs, daily activities, and other needs related to information, social, spiritual, dental, communication, sexual, financial aspects, and access to care [8]. Furthermore, its contribution to value-based healthcare for patients with HNC has been demonstrated through a randomised clinical trial in the UK, showing it to be a low-cost intervention that can improve patient satisfaction, multidisciplinary referrals, and the detection of otherwise overlooked issues [7]. The University of Washington Quality of Life questionnaire (UW-QOL) was used in conjunction with PCI to measure various aspects of health-related quality of life over the past week [9,10]. The UW-QOL has been translated and validated across several languages, including Turkish, Chinese, and Portuguese, demonstrating its adaptability and cross-cultural relevance [11,12,13]. Also, its Arabic version demonstrated adequate reliability (test–retest and internal consistency) and construct validity among a cohort of HNC patients in Morocco [9].

There remains little known about patient-reported needs and concerns in Arabic-speaking patients with HNC. PCI-HN was previously translated into Chinese, Dutch, French, German, Greek, Hindi, Malay, Mandarin, Serbian, Spanish, Polish, Portuguese, Tamil, and Urdu. However, no valid Arabic translation is currently available. Addressing cultural and linguistic differences among patient populations globally can improve the understanding of how patients perceive symptoms and express their concerns [14]. It is also mandatory to maintain personalised patient-clinician communication and education [7,14]. The present work aimed to translate and assess the reliability (test–retest and internal consistency) and validity (content, face, and construct) of the Arabic versions of the PCI for patients with HNC.

## 2. Materials and Methods

Patients attending the outpatient oncology clinics at King Saud University Medical City between March 2022 and January 2024 were enrolled in the study. A convenience non-random sampling method was used to identify potential participants aged 18 years or older with pathologically confirmed HNC diagnoses, who had received HNC treatment, and who were native Arabic-speaking patients [15,16]. Exclusion criteria included those younger than 18 years of age and who did not receive HNC treatment, had severely debilitating diseases, and could not read and understand the Arabic language.

The study investigators included an oral medicine consultant involved in the multidisciplinary HNC team (A.M.A.), a medical oncologist with a focus on head and neck cancers and provided care for these patients (S.A.F), two medical interns who were involved in oncology rotations (A.F.A. and B.A.A.) and a maxillofacial surgeon who had experience in patient concerns related to HNC (S.N.R.). Each participant was recruited after their routine care visits to a medical oncology clinic. At the end of the consultation, two study investigators (A.F.A. and B.A.A.) provided the patients with written information about the study. Those who agreed were recruited in the same or next care visit per their wishes. Potential participants received information about completing the electronic version of the questionnaires. After signing the informed consent form, the recruited participants completed the tablet-based information independently and reported no difficulties or technical issues.

### 2.1. Translation and Cross-Cultural Adaptation

The Arabic PCI (Ar-PCI-HN) was adapted from the original English versions using the forward-backwards translation method, in line with the principles of good practice for translating and culturally adapting patient-reported outcome measures (PROMs) [13,17]. Two bilingual study investigators conducted forward translation, two performed translation reconciliation, two addressed synthesis and modifications, and then two independent translators performed back translation into English. A panel of study investigators reviewed all versions for conceptual equivalence, harmonisation, and cultural appropriateness, as well as proofreading (Table 1) [17,18].

### 2.2. Content and Face Validity

The content validity was assessed by six clinicians, comprising three oral medicine specialists and three medical oncologists, all with more than 10 years of relevant experience in HNC care. Questions to ensure that all Ar-PCI-HN items are relevant to the patient population, the area of interest [e.g., the concerns of patients that they would like to discuss during their consultation], the context of interest [e.g., to address a wide variety of problems, assist direct outpatient appointments, and advance interdisciplinary therapy], and whether each response option is appropriate. Clinicians were also asked about comprehensiveness by asking, ‘Are all key concepts related to patient concerns included?’ (Supplementary File S1) [19,20,21]. All six clinicians viewed the items as comprehensive, appropriate, and relevant to the patient population and the area/context of interest. However, they had demonstrated specific comments and suggestions for the initial version (Table 2).

Additionally, cognitive debriefing and face validity were initially assessed through semi-structured interviews with six patients to evaluate relevance, comprehensiveness, and comprehensibility [17,20,21]. After reading the electronic version of Ar-PCI-HN using touch-screen technology [22], all noted that items were related and comprehensive. Although five of them noted a low understanding of the meaning of physical and functional well-being, attractiveness to a romantic partner, and were unsure about the roles of social workers and financial counsellors. After revisions, another group of eight patients found all revised items were indeed relevant and easy to comprehend (Table 3).

### 2.3. Construct Validity

Construct validity was examined using Spearman’s rank correlation coefficient (ρ) to assess associations between Ar-PCI-HN and the Ar-UW-QOL questionnaire (v 4.0) [9]. Based 4 or 5-point scale, this version included (1) items related to activity, anxiety, appearance, chewing, mood, speech, shoulder, recreation, saliva, swallowing, taste and pain; (2) a question to indicate up to 3 important symptoms to the patient of the previous items; and (3) global questions to rate the health-related quality of life over the past month, past week and overall [23]. Its Arabic translation was validated in a head and neck cancer patient cohort and demonstrated a good internal consistency (Cronbach’s α = 0.82) and excellent test–retest reliability (Intraclass correlation coefficient = 0.098) [22].

### 2.4. Statistical Analysis

Data were analysed using IBM SPSS (v.25). Internal consistency, which measures the interrelatedness within a multi-item instrument, was assessed using Cronbach’s alpha (α), with values ≥0.70 indicating acceptable reliability [19,24]. Assessments by Cohen’s Kappa (κ) for nominal variables were performed to assess the test–retest reliability of Ar-PCI-HN between the first and second completions by participants over 7–14 days [19]. These were interpreted based on the level of agreement as slight (<0.20), fair (<0.40), moderate (<0.60), substantial (<0.80), and almost perfect (>0.80) [25].

For the ordinal variables, the intraclass correlation coefficients (ICCs) were used to assess the test–retest reliability of the Ar-UW-QOL [19]. ICCs were interpreted as poor (<0.5–0.75), moderate (<0.75), or good-excellent (>0.75) [26]. Statistical significance was set at a *p*-value of 0.05 or less.

Ethical approval was prospectively approved by the Institutional Review Board of King Saud University [Ref: 23/0221/IRB, date: 14 February 2023]. All study participants read the study information sheet and signed the informed consent form, indicating their participation and consent for publication.

## 3. Results

The records of 113 patients were initially screened for eligibility. After excluding 75 records for patients awaiting HNC treatment and those in palliative care, 38 patients (males = 19, females = 19) with a mean age of 50 ± 16.13 years (range, 18–90 years) were invited and agreed to participate following their routine clinical care visits. Regarding their educational attainment, 22 had a high school education or less, 14 had a bachelor’s degree, and 2 had postgraduate degrees. They were mainly retired or unemployed (n = 24), with the remaining being either employed (n = 12) or students (n = 2). Regarding tobacco use and alcohol drinking, 29 of them (76%) indicated that they never smoked, 9 were previous tobacco users, and none indicated present or previous alcohol drinking.

**Figure 1:**
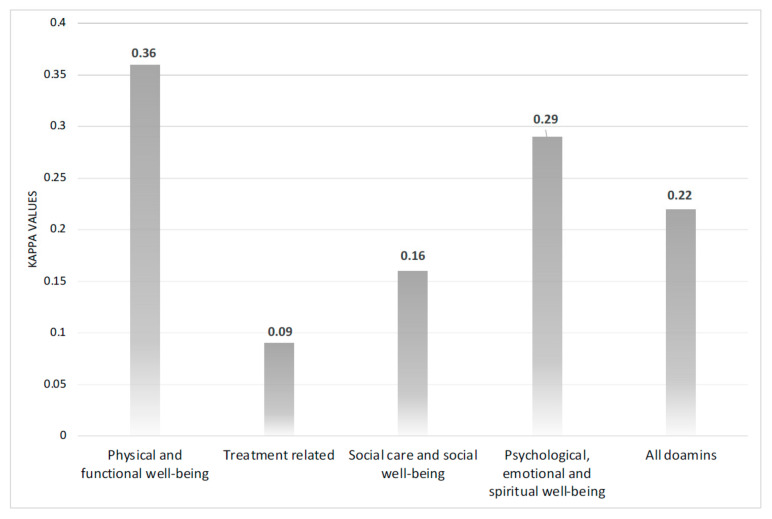
The Cohen’s κ coefficients (test–retest) for Ar-PCI-HN domains.

The Ar-PCI-HN demonstrated acceptable internal consistency, with a Cronbach’s α of 0.723; all items exceeded the cut-off for acceptable internal consistency reliability (α = 0.71). These included physical and functional well-being (α = 0.72), treatment-related (α = 0.72), social care and social well-being (α = 0.72), and psychological, emotional, and spiritual well-being (α = 0.72). Its test–retest reliability between the first and second completions by 31 of the 38 patients was fair (average κ = 0.22). Notably, the highest and lowest agreement was observed for physical and functional well-being (κ = 0.36) and treatment-related items (κ = 0.09), respectively (Figure 1).

The Ar-UW-QOL demonstrated strong internal consistency, with a Cronbach’s alpha of 0.753 and good test–retest reliability, as indicated by an ICC of 0.753. Domain-level analysis revealed that the most affected areas were saliva, swallowing, and appearance. Approximately 48% of participants reported dysfunction in at least one UW-QOL domain, 24% in two or more domains, and 9% in four or more. Mean composite UW-QOL scores indicated moderate impairment across most domains. Construct validity analysis revealed a weak, non-significant positive correlation between Ar-PCI-HN and Ar-UW-QOL total scores (ρ = 0.296, *p* = 0.119), suggesting these instruments capture overlapping but distinct constructs. The final version of the Ar-PCI-HN is available online (Supplementary File S2).

## 4. Discussion

This study represents the first attempt to translate, culturally adapt, and preliminarily test the internal consistency of Ar-PCI-HN in a cohort of patients with HNC. The results demonstrated that Ar-PCI-HN is a helpful tool for Arabic-speaking HNC populations. The internal consistency of the Ar-PCI-HN (α = 0.723) aligns with its English version (α = 0.76) [5], while the Brazilian Portuguese version demonstrated an alpha of 0.78 [6]. Similarly, the Ar-UW-QOL’s internal consistency (α = 0.753) is comparable to other versions, including the Turkish (α = 0.74) [13], Chinese (α = 0.79) [12], and Hindi (α = 0.75) [27], confirming its acceptable psychometric properties across different patient populations. Furthermore, the overall fair agreement between both completions by participants might be explained by the over-time changes in perceived needs and concerns in cancer care.

Clinically, the Ar-UW-QOL revealed that saliva, swallowing, and appearance were the most affected domains, as observed in another clinical cohort of post-radiotherapy HNC survivors [28]. Similar findings were noted in a Brazilian patient cohort, emphasising the tool’s sensitivity in detecting common treatment-related sequelae [29].

The relatively weak correlation between Ar-PCI-HN and Ar-UW-QOL (ρ = 0.296) confirms their complementary roles. While the UW-QOL quantifies functional impairment, the PCI identifies broader and more subjective concerns, such as emotional distress and family impact. A large UK randomised trial demonstrated that routine use of the PCI in outpatient consultations enhanced communication and identified concerns not typically captured by structured instruments [7]. Similarly, a study in Brazil confirmed that the PCI identified unmet psychosocial and practical needs in patients that would have otherwise gone unaddressed.

A significant negative correlation was found between the Ar-UW-QOL and the Xerostomia Inventory (ρ = –0.434), underscoring the burden of xerostomia on post-treatment quality of life. These findings are consistent with validation work in China and Turkey, where xerostomia and swallowing dysfunction were among the top concerns affecting quality of life (QOL) [12,13].

The results underscore the importance of implementing culturally adapted PROMs in Arabic-speaking clinical settings. In the Middle Eastern countries, where cancer incidence is projected to increase substantially [2,3], the need for patient-centred, culturally competent care is urgent. It also highlights the importance of considering diverse cultural and social interpretations across different patient populations (e.g., items related to social and religious welfare and financial counselling). The perceived importance of such topics might be less desirable to discuss with clinicians in a Western population but rather considered essential among those in the Arabic world [30,31]. For instance, decisions to own health in cancer care (e.g., palliative and end-of-life care) in Arabic countries are often affected by religious beliefs where a disease might be seen as a natural fate or indeed faith/patience testing and penance for sins [32]. Therefore, addressing these spiritual/cultural underpinnings with appropriate counselling might facilitate HNC diagnosis acceptance and early management [32,33]. This also explains why the related AR-PCI-HN followed cultural and religious views compared to its original version, while the measured construct in both versions remains similar (e.g., ‘Spiritual/religious aspects’ was adapted as ‘religion’).

PROMs, such as the PCI and UW-QOL, support early symptom detection, improved multidisciplinary referrals, and patient empowerment, all of which contribute to better treatment experiences and outcomes. Furthermore, integrating the PCI into routine care supports multidisciplinary communication, enhances psychological screening, and improves consultation quality without extending visit time [7,21]. The PCI has also been shown to enhance the quality of life when used consistently across follow-ups.

There remain little known related to access healthcare services in the Arabic countries due to sometime limited resources, increased out-of-pocket healthcare cost and insufficient expenditure for clinical services concerning cancer screening, surgical and chemoradiotherapy interventions and rehabilitation [34,35]. The is also a notable lack of clinical research that ascertaining the patient experiences and needs through the cancer journey, including what they wish to discuss with their care providers in short and perhaps overwhelming cancer services [36,37,38]. The cultural, religious, and social underpinnings are profound determinants of patient concerns in the Middle East and North Africa, where around half a billion Arabic speakers live [39]. Thus, Ar-PCI can be incorporated as a web-based tool as a hospital visit diary to help patients determine what they wish to address during their clinical visits with relevance. This can be available as a pre-visit checklist sent via phone text or email along with the appointment reminder. Then responses could be linked to the patient’s electronic medical records to support informed decisions about their care and to maintain tailored, patient-centred discussions. The Ar-PCI may also support an accurate referral process and resource allocation that addresses the patient’s care needs and maintains value-based healthcare services [5].

This study addresses a key gap in global oncology literature: the underrepresentation of Arabic-speaking patients in PROM validation. By offering validated instruments in a language patients can understand, this work provides a foundation for inclusive, high-quality care, enabling cross-cultural comparisons in survivorship research [40]. Additionally, using touch-screen/computerised questionnaires may be burdensome for individuals with low digital literacy or visual limitations [22]. However, this was not observed in the present study, which employed standardised settings and training for the study investigators (e.g., a quiet clinical waiting area, sufficient explanation of the study activities, appropriate timing for completing the questionnaires, and clarification of any unclear items). The relatively small sample size and single-centre design may limit the generalizability of the findings.

Further multicentre studies with larger and more diverse patient populations and clinical settings are needed to assess the over-time changes in perceived needs further to ensure that the presently limited test–retest (κ = 0.22) of Arabic PCI-HN is rather a reflection of clinical changes during the 7–14-day period of perceived needs than a structural limitation of the instrument. Additionally, further assessments are necessary to determine the criterion/construct validity (comparisons with the gold standard/instruments using similar constructs), responsiveness, and clinical interpretability of Ar-PCI-HN in different Arabic-speaking countries.

## 5. Conclusions

The Ar-PCI-HN is a culturally appropriate instrument with acceptable content and face validity, as well as internal consistency reliability, for assessing the multifaceted experiences of HNC survivors in Arabic-speaking countries. These assessments highlighted the essence and practical value of cross-cultural adjustments. There is, however, a need for further testing of other measurement properties to ensure their suitability for clinical practice, including identifying unmet needs, guiding individualised care, and enhancing survivorship outcomes.

## Figures and Tables

**Table 1:** Forward-backwards translations of the PCI-HN by the study investigators.

Original PCI	Backwards Translated Text Discrepancy
Physical and functional well-being	Physical and functional health *
Chewing/eating	Chewing *
Dental health/teeth	Oral hygiene *
Mobility	Ability to move *
Regurgitation	Reflux *
Sore mouth	Mouth ulcers *
Vomiting/sickness	Vomiting *
Social care and social well-being	Recreational and social well-being *
Dependents/children	Children *
Home care/District nurse	Home care *
Recreation	Relaxation *
Psychological, emotional and spiritual well-being:	Emotional, psychological, and mental well-being *
Intimacy	Sexual relations *
Sexuality	Sexual activity *
Spiritual/religious aspects	Religion *
Physiotherapist	Physical therapist *
Dietician	Nutritionist *
Oncologist/Radiotherapist	Oncologist/radiation oncologist *
Chaplain	Clergyman *
Speech (swallow) and language therapist	Speech (swallowing) and language specialist *
Social care and social well-being:	Social health and care *
Treatment related	Related to your treatment **
PEGtube	Feeding tube **
Carer	Caregiver **
Speech/voice/being understood	Speaking/voice/being understood **
Support for my family	Familial support **
Fear of the cancer coming back	Fear of cancer recurrence **
Fear of adverse events	Fear of side effects **
Clinical psychologist	Psychologist **

* Revised to reflect the original PCI items, semantic and conceptual meanings and cultural relevance/acceptability. ** No action performed.

**Table 2:** The clinicians’ responses to questions about Ar-PCI-HN items (content validity).

Study ID	Summary	Suggestion/s	Action/s
C01 ^1^	*All items are excellent, relevant, and comprehensive, except for the irrelevance of the social and religious welfare items during patients’ consultation*	*1-Adjust as needed* *2-Communicator should be there when patients fill out the questionnaire*	The items related to the social and religious welfare were revised for clarity
C02	*Some items are similar and should be revised,* e.g., *those related to fatigue, energy levels and movement, bowel and indigestion, as well as those related to oral dryness, drooling and saliva problems*	*Use fewer words in each Item*	Many of these items were measuring distinct concerns/aspects; thus, they cannot be omitted Also, it was not feasible to omit items related to cancer fear and other psychosocial aspects, as international and regional HNC studies frequently report those Wording and linguistic revisions were performed to match those used in daily conversations and were deemed suitable for the patient cohort Some of the items were kept due to their comprehensiveness (e.g., ‘head and neck pain’ rather than ‘oral pain’) Specifying
C03 ^1^	*1-Some words need clarification* *→* *advantages, energy levels, family support, financial support, leisure, social relationships, head and neck pain, intubation, orofacial pain specialist, and maxillofacial specialist* *2-Words with no values in the medical field, such as fear of cancer return, intimacy and love, mood, self-esteem, and attractiveness to a partner* *3-Regret to have the ‘treatment’ is not suitable* *4-Specify each cancer therapy type*	*1-‘Income’ rather than financial support from the government* *2-Use ‘oral pain’ rather than ‘head and neck pain’* *3-Revise how to eat food and wound healing* *4-Remove all items with no value because they have no value in the targeted population* *5-Specify the treatment as radiotherapy, chemotherapy, surgical, or hormonal*	
C04, C05 ^2^	*None*	*None*	No change
C06 ^2^	*A communicator is needed with the patients to fill it in an optimal way*	*As indicated*	No change

^1^ Allied to oral medicine, ^2^ Allied to radiation oncology. Abbreviations: C, clinician participant.

**Table 3:** The patient’s responses to questions about Ar-PCI-HN items (face validity).

Study ID	Feedback *	Action/s
P01	Difficulty understanding ‘physical and functional well-being’ and ‘well-being’	The items were revised by the study team for understandability and cultural relevance
P02	Difficulty understanding ‘the person’s attraction to their romantic partner’ and ‘social psychologist’	
P03	Difficulty understanding ‘physical and functional well-being’, ‘financial counsellor’, ‘advocate’, and ‘social worker’	
P04	None	None
P05	Difficulty understanding ‘physical and functional well-being’	
P06	None	None
After the first round of revisions		
P07–14 **	All items were easy to understand	None

* Expressed in the patient’s language (Arabic). ** The participant P14 noted that they find it difficult to recognise the relevance of items, as they had not yet encountered any adverse effects. Abbreviations: P, patient participant.

## Data Availability

Data is contained within the article or Supplementary Materials. The permission to use and translate the PCI-HN was obtained from the copyright holder.

## Supplementary Materials

The following supporting information can be downloaded at https://www.mdpi.com/article/10.3390/curroncol33010012/s1: Supplementary File S1: The content validity assessment by clinicians (n = 6), Supplementary File S2. The Arabic Patient Concern Inventory for Head and Neck [Ar-PCI-HN].

## Abbreviations

Ar-UW-QOL	University of Washington Quality of Life Questionnaire
HNC	Head and neck cancer
PROMs	Patient-reported outcome measures
PCI-HN	Post-treatment Patient Concerns Inventory for head and neck
Ar-PCI-HN	Arabic PCI-HN
