# Supplementary material for: Patient Concerns Inventory for Arabic Patients with Head and Neck Cancer: A Cross-Cultural Adaptation and Preliminary Validation
Source: Curr Oncol. 2025 Dec 24;33(1):12. doi: 10.3390/curroncol33010012 (PMC12839732; doi:10.3390/curroncol33010012)
Supplement: Supplementary file 1 [file curroncol-33-00012-s001.zip › Supplementary File S2.pdf]

Supplementary File S2. The Arabic Patient Concern Inventory for Head and Neck [Ar-PCI-HN].

The Ar-PCI-HN can be accessed at: https://livheadandneck.co.uk/patients/patient-concern-inventory/pci-translations
