# Supplementary material for: Patient Concerns Inventory for Arabic Patients with Head and Neck Cancer: A Cross-Cultural Adaptation and Preliminary Validation
Source: Curr Oncol. 2025 Dec 24;33(1):12. doi: 10.3390/curroncol33010012 (PMC12839732; doi:10.3390/curroncol33010012)
Supplement: Supplementary file 1 [file curroncol-33-00012-s001.zip › Supplementary File S1.pdf]

Supplementary File S1. The content validity assessment by clinicians (n = 6).

Validation of the Patient Concerns Inventory (PCI)

Study objectives

The present work aims to assess the content validity of the Patient Concerns Inventory (PCI) scale by expert clinicians.

Relevance

All items in a PROM should be relevant for the construct of interest within a specific population and context of use.

Instructions

Section 1. Please complete your background information.

Gender: □ Male □ Female

Speciality: □ Oral Medicine □ Oncology □ Others (please specify):_________

Years of experience as a specialist: □ 1-5 years □ 5-10 years □ > 10 years

Section 2. Please read the PCI scale and provide your opinion in the next section regarding the relevance and comprehensiveness of this scale for patients with head and neck cancer.

Please turn to the next page →

Section 2. Please answer the following questions about the PCI scale.

Question	Yes	No	If your answer is no, please mention which item(s) you would like to modify, add, or omit?
**Relevance**			
Is each item relevant to the patient population (i.e. patients with head and neck cancer and receiving radiotherapy)?			Item_____<br>Suggested change<br>_________________________<br>__________________
Is each item relevant to the area of interest (i.e. the concerns of patients that they would like to discuss during their consultation)?			Item_____<br>Suggested change<br>_________________________<br>__________________
Is each included item relevant for the context of interest (e.g. to address a wide variety of problems, assist direct outpatient appointments, and advance interdisciplinary therapy)?			Item_____<br>Suggested change<br>_________________________<br>__________________
Is each response option appropriate?			Item_____<br>Suggested change
**Comprehensiveness**			
Are all key concepts regarding the concerns of patients included?			Item_____<br>Suggested change<br>_________________________

Please add any other comments regarding the PCI scale.

____
